# French Law and Custom for English Visitors
*"French Law and Custom for the Anglo-Saxon: A Guide for Everyday Use." By Arthur S. Browne, Solicitor of the Supreme Court. Second edition, revised and enlarged. Price 2s. 6d. net. London: The Health Resorts Bureau, 27 Chancery Lane, W.C.


**Published:** 1907-05-25

**Authors:** 


### FRENCH LAW AND CUSTOM FOR ENGLISH VISITORS.*

Every year more and more English people go to France for health, pleasure, or business. Every year many of them come home grumbling at some misunderstanding or grievance, the result of imagining that English laws and customs should prevail everywhere. It may be stupid and benighted of foreigners to prefer their own ways to ours, but since it is so, we must e'en submit, or stop at home. But the French, foreigners though they be, have a wise proverb which says that "to know all is to forgive all," and perhaps if we understood their ways better we should not so often fall foul of them. Mr. Arthur Browne has compiled in a handy volume a great deal of information which should be of the greatest use to tourists or residents in France, or, indeed, to any who have dealings with Frenchmen either on this side of the Channel or the other. It conducts the Englishman in France literally from the cradle to the grave, for one of the earliest sections deals with the delicate question of naturalisation. It is well to know that the child even of British parents, if he is born in France, must, if he is domiciled in that country, make a declaration within the year following his twenty-first birthday that he wishes to decline French nationality, or he will be regarded as a Frenchman, and consequently will have to serve in the army, and in every other way fulfil the duty of a French citizen. Until he attains his majority he is regarded as French. Thus, as Mr. Browne points out, "it is quite conceivable that a man may, according to French law, be a Frenchman without knowing it."

### Landlord's Liability.

But the majority of English persons who go to France go there only as visitors, and many a one will be glad to learn just what are his rights when he takes up his abode in a hotel. In some hotels notices are hung up stating that rooms are held to be taken by the week, but the law holds that a room is taken only by the day, and a visitor cannot be made to pay for more days than he occupies it, unless he has entered into pension terms, by which he obtains board and lodging at a certain sum per week. But in the absence of such special arrangement he is regarded as a daily tenant. A hotel-keeper is responsible for his visitors' property, and if the latter's effects are stolen the proprietor must make the loss good, but in the case of money, or securities for money payable to bearer, which have not been entrusted to the landlord for safe keeping, the liability is limited to £40 sterling, except in the principality of Monaco, where the hotel proprietor's liability is unlimited. If, however, it can be shown that when a robbery has been committed the guest has been guilty of contributory negligence, the landlord may be exonerated altogether. It is useful also to know that a hotel-keeper has no claim for damages against a visitor who may develop infectious disease while staying at his hotel, but can only claim to be repaid any expense to which he may be put in disinfecting the rooms occupied by the sick person. If, however, the visitor is requested to leave, could do so without endangering his health, but refuses to go, the hotel-keeper may have a claim for the loss entailed upon him through the injury the presence of the disease will do to the reputation of his hotel. Further, in the case of a death, the hotel-keeper has no right to make any claim for the loss to the house, even though the fact of a death occurring may empty his hotel. He can, as in the case of sickness of a guest, claim to be recouped for the cost of putting the death-chamber into a proper sanitary condition in accordance with the requirements of the authorities, and for any actual damage. It is possible that the good feeling of friends and relatives may add a solatium for trouble given and injury to the business of the hotel, but this is purely an act of grace, and not a legal claim. It is curious to know that in many French towns it is customary to entrust the funerals of Roman Catholics to one undertaker, and those of Protestants to another. This division rather helps to simplify arrangements in the case of a death. Nurses who go abroad with patients would do well to consult those chapters of Mr. Browne's book which deal with sickness and death and the responsibilities entailed by these before the law. Occasions may well arise when such knowledge would be very useful to helpless and ignorant mourners who are neither able, nor in the humour, to investigate these matters for themselves.

### Register! Register!

The nurse would have a responsibility to herself also as a wage-earner. One of her first acts should be to register herself. By the law of August 8, 1893, all foreigners, male or female, who wish to earn money in France, must make themselves known at the Mairie in a provincial town, or at the Prefecture of Police at Paris, within eight days of arrival. They should have papers to prove their identity. For this, a passport and a certificate of birth or baptism is desirable, but failing these, a certificate of identity, delivered by the applying foreigner's Consul, will be accepted. The applicant must also submit to be vaccinated, or prove that the operation has been performed recently. When all these formalities have been gone through, the foreigner is granted a certificate of residence for which a payment of 2 francs 55 centimes (about 2s.) is charged. If the foreigner moves to another commune or district, the certificate must be endorsed at the Mairie of the new residence. It is important to know also that everyone who knowingly employs a foreigner who has not been duly registered is liable to a fine.

### The Marriage Law.

The risks run by an Englishwoman who marries a Frenchman without complying with all the regulations of French law as to the consent of parents or near relatives, has often furnished a subject for novelists, and unfortunately romances on this topic are too often founded on fact. There is a great deal to be said for the French law; at least it prevents boys and girls making rash matches of which they, as often as not, repent before long. But it is well to know just what the regulations for a duly legal marriage are. This, together with useful facts about will-making, intestacy, the law as to the payment of the rent of houses, notice to quit them, engaging and dismissing of servants, married women's property, the purchase of real estate, and even the French law with regard to limited companies, will be found in Mr. Browne's book, which also contains in an interesting appendix valuable information as to travelling in France, French weights and measures, and the comparative cost of living at home and abroad. Indeed, this is a *vade-mecum* both for English travellers, and for English residents among our neighbours on the other side of the Channel.

* "French Law and Custom for the Anglo-Saxon: A Guide for Everyday Use." By Arthur S. Browne, Solicitor of the Supreme Court. Second edition, revised and enlarged. Price 2s. 6d. net. London: The Health Resorts Bureau, 27 Chancery Lane, W.C.

